# Factors that Affect the National Student Performance Examination Grades of Brazilian Undergraduate Medical Programs

**DOI:** 10.3205/zma001155

**Published:** 2018-02-15

**Authors:** Toufic Anbar Neto, Patricia da Silva Fucuta Pereia, Mauricio L. Nogueira, Jose Maria Pereira de Gody, Airton C. Moscardini

**Affiliations:** 1 Ceres Medical School (Faculdade Ceres - FACERES), Department of Medical Education, São José do Rio Preto, Brazil; 2 Ceres Medical School (FACERES), Department of Gastroenterology, São José do Rio Preto, Brazil; 3 São José do Rio Preto Medical School (Faculdade de Medicina de São José do Rio Preto - FAMERP), Health Sciences Graduate Program, São José do Rio Preto, Brazil; 4 São José do Rio Preto Medical School (Faculdade de Medicina de São José do Rio Preto - FAMERP), Department of Cardiology and Cardiovascular Surgery, São José do Rio Preto, Brazil; 5 São José do Rio Preto Medical School (Faculdade de Medicina de São José do Rio Preto - FAMERP), Department of Pediatrics and Pediatric Surgery, São José do Rio Preto, Brazil

**Keywords:** independent medical assessment, medical school, medical education

## Abstract

**Background: **The Brazilian National Student Performance Examination - ENADE is an instrument used to measure the quality of undergraduate courses. The identification of factors that influence the result of this examination can contribute to providing support necessary to improve the quality of medical courses. The purpose of this work was to evaluate the factors that affect the National Student Performance Examination grades of Brazilian undergraduate medical programs.

**Methods:** Factors that influenced the 2010 ENADE grades of 100 undergraduate medical programs were studied. Data collection was performed using public databases. Academic and healthcare infrastructure variables were investigated. The data analysis was based on the performance of the medical programs on the 2010 ENADE, whereby the programs were divided into two groups: ENADE 1-2 (unsatisfactory grade) versus ENADE 4-5 (satisfactory grade).

**Results: **One hundred schools were included in this analysis. In the univariate analysis the university variables (p=0.037), public institution (p<0.001), lower number of openings per course (p=0.036), lower number of specialist professors (p=0.003) and higher number of doctors (p=0.010), *strictu sensu* post-graduation program (p<0.001), higher course lifetime (p<0.001) were associated to best results in ENADE. In the multivariate analysis of logistic binary regression four variables remained independently associated to a better performance in ENADE: public institution (OR 9.9; 95%CI 1.03 to 95.5), lower number of openings per course (OR 0.984; 95% CI 0.969 to 0.999), *strictu sensu* post-graduation program (OR 8.189; 95% CI 1.459 to 45.971) and longer course lifetime (OR 1.058; 95% CI 1.013 to 1.105).

**Conclusions: **The satisfactory score of this evaluation (ENADE 4-5) was associated to the public administration category of higher degree institutions, lower number of openings offered per course, the presence of a* strictu sensu* and longer course lifetime.

## Zusammenfassung

**Hintergrund: **Das nationale brasilianische Examen zur Erfassung der Studentischen Leistung – ENADE (Exame Nacional de Desempenho do Estudante) untersucht die Qualität medizinischer Grund- bzw. Bachelorstudienprogramme. Die Identifikation der Faktoren, die die Ergebnisse dieses Examens beeinflussen, kann dazu beitragen, die notwendige Unterstützung zur Verbesserung der Qualität medizinischer Ausbildungsprogramme bereitzustellen. Das Ziel dieser Arbeit war die Evaluation der Faktoren, die einen Einfluss auf die Ergebnisse brasilianischer Medizinstudierender im ENADE haben.

**Methoden:** Untersucht wurden alle Faktoren, die 2010 die ENADE-Ergebnisse von 100 medizinischen Studiengängen bedingt haben. Die Daten wurden aus öffentlichen Datenbanken erhoben und im Hinblick auf strukturbezogene Variablen im Bereich des Hochschul- und Gesundheitswesens untersucht. Die Datenanalyse basiert auf den Ergebnissen der medizinischen Studienprogramme beim ENADE 2010, wobei die Studienprogramme in zwei Gruppen aufgeteilt wurden: ENADE 1–2 (Studienprogramme mit unbefriedigenden Ergebnissen) gegenüber ENADE 4–5 (Studienprogramme mit befriedigenden Ergebnissen).

**Ergebnisse: **Die Analyse umfasste die Untersuchung von 100 Ausbildungsstätten. Bei der univariaten Analyse zeigte sich eine Korrelation zwischen besseren ENADE-Ergebnissen und den Variablen Universität (p=0,037), staatliche Ausbildungsstätte (p<0,001), geringes Anzahl an Studienplätzen (p=0,036), weniger Lehrende mit einem „Spezialisten“-Titel (Especializacao) (p=0,003) und mehr Lehrende mit einem Doktortitel (p=0,010), *stricto sensu* Post-Graduiertenprogramm (cursos de pós-graduação *stricto sensu*) (p<0,001), sowie längeres Bestehen des Studienprogramms (p<0,001). Bei der multivariaten Analyse der binären logistischen Regression wurde gezeigt, dass vier Variablen lose mit besseren ENADE-Ergebnissen korrelieren: staatliche Ausbildungsstätte (OR 9,9; 95%CI 1,03 bis 95,5), geringes Angebot an Studienplätzen (OR 0,984; 95% CI 0,969 bis 0,999), *stricto sensu* Post-Graduiertenprogramm (cursos de pós-graduação *stricto sensu*) (OR 8,189; 95% CI 1,459 bis 45,971) und längeres Bestehen des Studienprogramms (OR 1,058; 95% CI 1,013 bis 1,105).

**Schlussfolgerung: **Das Erreichen eines befriedigenden Ergebnisses bei diesem Examen (ENADE 4–5) wurde mit der staatlichen Administration von Hochschulen, dem geringeren Angebot an Studienplätzen pro Studienprogramm, dem Angebot von *stricto sensu* Post-Graduiertenprogrammen sowie dem längeren Bestehen der Studienprogramme in Verbindung gebracht.

## Introduction

The evaluation of undergraduate medical programs is considered controversial for many higher education institutions (HEIs). The first evaluation of medical schools with global significance was the study conducted by Abraham Flexner in the United States in 1910, which became known as the Flexner Report [1]. Used as a reference worldwide, this study resulted in significant changes in medical education. More than 100 years after its publication, the report continues to generate controversy. The strength of Flexner’s report is the result of the comprehensive nature of it evaluation in numerical terms, its emphasis on a scientific foundation and, mainly, that it was directed primarily toward society at large [2].

The evaluation of medical programs may be performed by federal agencies [3], [4] and, as in the United States, by private consortiums that involve groups of universities. The evaluations are performed to assess such programs individually and to compare them with those of other participants that participate in the same evaluation process [5], [6], [7]. The instruments used to evaluate medical programs include tests to measure the programs [4], tests to measure proficiency in practicing the profession [8] and nonspecific educational tests [9], [10].

Since the 1960s, the Brazilian federal government and the Ministry of Education (Ministério da Educação - MEC) have been more concerned with evaluating HEIs as a whole, and several public policies have been developed with this goal [11].The educational policies implemented during the 1990s increased the visibility of the higher education evaluation process.

The Brazilian Educational system comprises 3 types of higher education institutions (IES): College (small size), university center (intermediate size) and university (large size). The amount of courses is what defines each one of them (a minimum of 8 for university centers and 12 for universities), the administrative structure (Universities and university center have a department of undergraduate studies), post-graduation programs (only for universities) and titles for the team of professors (Doctor’s degrees for more than 30% for universities). The system is divided in public IES (managed and defrayed by the government, with no cost for the student) and private (generally profit-driven institutions, paid by the students).

The process of evaluating educational programs progressed with the creation of the National System of Higher Education Evaluation (Sistema Nacional de Avaliação da Educação Superior - SINAES) by Law no. 10,861, April 14, 2004 [12]. The instruments used by SINAES include institutional self-evaluation, external evaluation by members of the community in which the HEI is located, undergraduate program evaluation by committees appointed by the National Institute of Educational Studies and Research (Instituto Nacional de Estudos e Pesquisas Educacionais - INEP), the annual census of higher education and the National Student Performance Exam (Exame Nacional de Desempenho de Estudantes - ENADE). The purpose of SINAES is to obtain a general overview of the quality of the country’s programs and HEIs to guide public and institutional policies [12].

ENADE is the most well-known and widespread instrument, and it is an important part of evaluating the quality of undergraduate programs. ENADE aims at supervising the learning process and the academic performance of students regarding the predicted program contents in the medical school curricular guidelines for the course. It evaluates student skills, competencies and performance with regard to the program’s content as established by the National Curriculum Guidelines (Diretrizes Curriculares Nacionais - DCN) for each undergraduate subject area [13]. The examination for each subject area is given every three years [14]. ENADE participation is mandatory for all students who by the final day of examination enrollment have completed at least 80% of the minimum credit hours required for the subject area under evaluation [15]. The examination has three parts: questions that measure general education, specific components and the perception of the test [16]. The grades range from 1 (poor performance) to 5 (best performance), and a grade between 3 and 5 is considered satisfactory [15]]. All IES institutions expect achieving grades 4 and 5 in ENADE, since they broadly use this feature to attract new students.

Research on the factors that affect the ENADE grades of Brazilian undergraduate medical programs is necessary because no studies with this focus were found in the literature. Identifying the factors that influence performance on this examination may contribute to providing necessary support for improving the quality of medical programs and therefore ENADE grades. 

The objective of this study was to evaluate the factors that affect the ENADE grades of undergraduate medical programs considering demographic, academic, educational and healthcare infrastructure variables. 

## Methods

The factors that influenced the 2010 ENADE grades of Brazilian undergraduate medical programs were studied. Of a total of 136 programs, 100 (73.5%) were investigated. Because this study used public data available online and through other official federal government sources, it was not necessary to submit the project to the Research Ethics Committee.

The lack of a ENADE grade because of a medical program’s voluntary non-participation or because a program had not yet produced students qualified to take the examination were considered to be the exclusion criteria. 

The data collection was performed in public domain databases available on the websites of the MEC, the Ministry of Health and the Brazilian Institute of Geography and Statistics (Instituto Brasileiro de Geografia e Estatística - IBGE). Spreadsheets provided by the INEP were also used.

Ten academic, educational and structural variables were analyzed. Academic and educational variables by category included academic organization (university x non-university), administrative category (public x private), teaching-learning methodology (active x traditional), post-graduation program (with strictu sensu x without strictu sensu). Academic and educational categories of numeric nature comprised the number of vacancies each course can offer according to the Department of Education, the total amount of hours in the course, number of professors according to titles (specialists, masters or doctors), total number of professors, course lifetime in years. The health infrastructure variable of numeric nature studied was total number of beds/1000 inhabitants.

The analysis of the demographic, academic, educational and healthcare infrastructure variables was performed after categorizing the medical programs according to their performance on the 2010 ENADE. The programs were divided into two groups: ENADE 1-2 (unsatisfactory grade) versus ENADE 4-5 (satisfactory grade). The grade of 3, which was achieved by 36 medical programs, was not analyzed because it represents an intermediate grade. 

The descriptive analysis included absolute and relative frequencies for categorical variables and median and variation for continuous variables. Comparisons between the two groups (ENADE 1-2 versus ENADE 4-5) were performed using the Mann-Whitney U test for nonnormally distributed continuous data, and the chi-square test or Fisher’s exact test for categorical data (when appropriate). Variables that were found to be predictors of ENADE 4-5 in the univariate analysis were also included in a multivariate logistic regression model (stepwise with backward elimination method) and the results were presented as odds ratios with a 95% confidence interval. Statistical analyses of the data were performed using the IBM-SPSS Statistics version 24 (IBM Corporation, NY, USA). All tests were two-tailed, and P < 0.05 was considered statistically significant.

## Results

One hundred and thirty-six schools were analyzed by ENADE in 2010. Table 1 (Tab. 1) demonstrates medical schools’ performance according to ENADE classification.

In order to perform a comparison analysis, all IES were categorized into two groups: ENADE 1-2 (unsatisfactory: 25 schools) and ENADE 4-5 (satisfactory: 75 schools), therefore, by excluding score 3, which represents an intermediate score. Thus, 100 IES were included in this analysis. Table 2 (Tab. 2) demonstrates their fundamental features.

### Univariate comparative analysis between groups ENADE 1-2 and ENADE 4-5

Regarding the academic organization, we observed that universities have achieved a higher proportion in the satisfactory score (ENADE 4-5) comparing to non-university IES (p=0.037). In addition, public institutions also presented better performance comparing to private institutions (p<0.001). Number of openings per institution also was associated to these results and were inversely proportional to students’ performance. 

On the other hand, course hours and learning-teaching methodology showed no effect on results for this evaluation. Table 3 (Tab. 3) demonstrates the comparison of various academic variables to IES performance in ENADE 2010.

#### Healthcare infrastructure

The total number of beds where the course is present was also observed to present no influence on students’ performance. For ENADE 1-2 group, the median for the total/1000 number of beds was 3.41 (minimum=0.69; maximum=9.13) and for ENADE 4-5 group it was 3.95 (minimum=1.86; maximum=15.69), *p*=0.120.

#### Multivariate analysis on variables associated to students’ performance.

All seven variables associated to ENADE 2010 results were included in the multivariate analysis model of binary logistics regression. Four variables were observed to remain as independent factors: IES public administration category, less number of openings offered by the

satisfactory score in the evaluation (ENADE 4-5). Table 4 (Tab. 4) shows the results of this analysis expressed in odds ratio with a confidence interval of 95%.

## Discussion

This study evaluated the factors that affected the 2010 ENADE grades of undergraduate medical programs in all of Brazil’s regions. The factors that positively affected ENADE grades include being located in a university, a longer period of existence, a smaller number of authorized openings for students, a predominance of faculty members with master’s degrees and doctorates, the existence of *sensu stricto* graduate programs, being part of a public HEI and a larger number of total hospital beds. 

Regarding the classification of the medical programs according to their academic organization, 70.6% belonged universities, although this type of institution represents only 8% of Brazilian HEIs [17].

Of the programs located in universities, 82.9% obtained a grade of 4 or 5, whereas only 60% of the programs located in faculties obtained these grades. This outcome may result from the complexity of universities, which are institutions that should offer undergraduate and graduate programs, have a largely full-time faculty with advanced degrees, have consolidated and well-rated research centers, produce high-level scientific publications and possess extensive infrastructure [18], [19]. Additionally, to be classified as a university, an HEI must have a higher level of professionalization, which may positively affect the quality of its programs.

Regarding the duration of existence of the Brazilian medical programs, all of them allow a minimal completion of their credit hours requirement in six years. The presence of programs that have only existed for five years is because the ENADE is mandatory for students who have completed 80% of a program’s minimum credit hours [15]. As for the program’s duration of existence on the ENADE date, programs in the ENADE 4-5 group had a duration of existence approximately 5 times longer than those in the ENADE 1-2 group. 

 A program’s duration of existence affects the grade. If the classification rankings of world universities are analyzed, the best universities are among the oldest HEIs. The 10 universities ranked highest in the Academic Ranking of World Universities (ARWU) classification of Shanghai’s Jiao Tong University are more than 100 years old [http://www.timeshighereducation.co.uk/world-university-rankings/2013-14/world-ranking]. An element of institutional maturity results from complex long-term processes and a reputation that attracts the best professors and students, thus perpetuating the characteristics of excellence [20]. The development of a strong culture of excellence is the result of a gradual process, and its consolidation occurs over several decades and, at times, centuries. A medical program’s duration of existence cannot be considered to guarantee academic excellence. Factors such as adequate funding, modern administration, talent in research and institutional autonomy are equally or more important than the duration of existence [http://www.timeshighereducation.co.uk/world-university-rankings/2013-14/world-ranking].

In regard to the number of openings for students, the median of the ENADE 1-2 group was 100 openings, which surpasses the ENADE 4-5 group (median=80). The difference between the groups may be explained by the assets that a medical program requires, such as material resources, professors and hospital beds, which a program with more openings will not always possess in sufficient numbers. In Brazil, the process of creating medical programs occurred in several stages [20], and there was no consistent policy to regulate the number of openings available for students. Class size as a determining factor in the quality of teaching is controversial. Some believe that when a professor works with smaller classes, more individualized attention and an improvement in the quality of student learning are facilitated. In an intermediary and relatively ambiguous field, educational administrators, without disagreeing with the benefits of smaller classes, emphasize the budgetary constraints that result from this initiative. In addition, there are educational researchers for whom existing evidence does not support the assertion that smaller classes result in better learning [21], [22], [23].

Regarding the degrees held by a program’s faculty, there is a predominance of specialists in the ENADE 1-2 group and master’s and PhDs in the ENADE 4-5 group. In Brazil, there are two types of Post-graduation: “lato sensu”, which provides the specialist title and may be offered by all types of higher education institutions, and “strictu sensu”, which provides a Master’s or Doctor’s degree, and can only be offered by Institutions accredited by the Secretary of Education.

There is greater difficulty in HEIs that lack teaching staff training centers [24]. Conversely, public universities preferentially admit titled professors. These two extremes may explain the large disparity between the minimum number of faculty members with doctoral degrees (n=0) and the maximum number (n=371). In Brazil, the title of specialist, which is granted by societies of medical specialties and the National Commission of Medical Residency (Comissão Nacional de Residência Médica - CNRM), is equivalent to the title of a *sensu lato* specialist. Thus, there is a greater availability of professors with this degree [25]. A better qualified faculty has a higher intellectual and scientific level and can positively influence teaching quality [26] ,[27].

The total credit hours of the ENADE 1-2 group exhibited a median value of 8,298 hours, which was slightly lower than the ENADE 4-5 group (median of 8,400 hours). The National Council for Education (Conselho Nacional de Educação - CNE)’s Resolution no. 2, June 18, 2007, requires a minimum of 7,200 credit hours [28]. The HEIs have autonomy in determining their curriculum and their total number of credit hours [29]. The instances in which the number of credit hours is lower than the number established by law are all public universities. There is a slight difference in favor of the ENADE 4-5 group. However, how credit hours are used is more important than the total number of credit hours. If a faculty lacks good qualifications, a medical program’s total number of credit hours is irrelevant. Thus, other factors, such as the quality and diversity of teaching and learning activities and, primarily, the quality of the faculty, are more relevant than the number of credit hours [30].

The majority of the medical programs (93.4%) with *sensu stricto* health graduate programs on the same campus as the undergraduate program obtained a grade of 4 or 5. The majority of programs (53.8%) without these graduate programs obtained a grade of 1 or 2. We consider knowledge production, the training of qualified faculty absorbed by the institution itself and scientific production to be likely reflections of the existence of these graduate programs, whose primary purpose is to stimulate research and train the teaching staff [31].

Of the medical programs affiliated with public HEIs, 96.3% obtained a satisfactory grade, whereas this proportion was 50.0% in private HEIs. This outcome occurred because public institutions have a better structure, larger investment in research and restrictions on hiring unqualified personnel. Nevertheless, it is worth noting that the expansion of medical programs is currently concentrated in the private sector. In this sector, the MEC demands higher quality [32].

Of the medical programs that have adopted the traditional teaching method, 76.3% obtained a grade of 4 or 5, and this value was 70.0% for programs with the active method (AM). Contrary to what is observed in the existing literature, we do not believe there is a significant difference between these methods [33]. We believe that because of its short duration of existence in Brazil and the number of medical programs that have adopted the AM, the 2010 ENADE grade was satisfactory. 

Since the DCN’s publication, many medical schools have been reorganizing their curriculum and seeking new teaching methods, such as AM. In Brazil, the most significant methods are Problem-Based Learning (PBL) and problematization based on the “Maguerez arch” [34]. The focus is on primary healthcare, whereby the teaching and learning spaces should include the Basic Health Units (Unidades Básicas de Saúde - UBS), particularly those that serve the Family Health Program (Programa de Saúde da Família - PSF), to ensure comprehensive care and an interdisciplinary view of the medical program. 

Regarding the total number of hospital beds in the municipalities in which the medical programs are located, the ENADE 1-2 group exhibited a median of 1,461 beds, which was less than the ENADE 4-5 group (2,253 beds). Although this variable is not consistently used in the evaluation instrument for medical programs [16], we believe that this variable is important because diversified learning and teaching scenarios enrich the training of undergraduate students.

After following a series of ENADE grades, we suggest expanding the variables under study to consolidate the mechanisms for evaluating medical programs. This study identified strategic aspects for reflecting on the approaches to be taken by regulatory agencies and medical program administrators. Starting from the use of these evaluation parameters, a possible proposal for standardizing evaluation systems in different countries can be developed.

## Conclusions

The factors that positively affected the ENADE grades of medical programs included a longer period of existence, a smaller number of authorized openings for students, existence of* stricto sensu* graduate programs and being part of a public HEI.

## List of abbreviations

- ENADE: National Student Performance Examination
- HEIs: higher education institutions
- MEC: Ministry of Education
- SINAES: National System of Higher Education Evaluation
- INEP: National Institute of Educational Studies and Research
- DCN: National Curriculum Guidelines
- IBGE: Brazilian Institute of Geography and Statistics
- ARWU: Academic Ranking of World Universities
- CNRM: National Commission of Medical Residency
- CNE: National Council for Education
- PBL: Problem-Based Learning
- UBS: Basic Healthcare Units
- PSF: Family Health Program; AM: active method

## Authors’ contributions

TAN contributed to conception, design and acquisition of data as well as the analysis and interpretation of the data; PSFP contributed to the analysis and interpretation of data and was involved in drafting the manuscript and revising it critically for important intellectual content; MLN contributed to conception design and acquisition of data and gave the final approval of the version to be published; ACM contributed to the drafting of the manuscript and revising it critically for important intellectual content and gave the final approval of the version to be published.

All authors read and approved the final product.

## Competing interests

The authors declare that they have no competing interests. 

## Figures and Tables

**Table 1:**
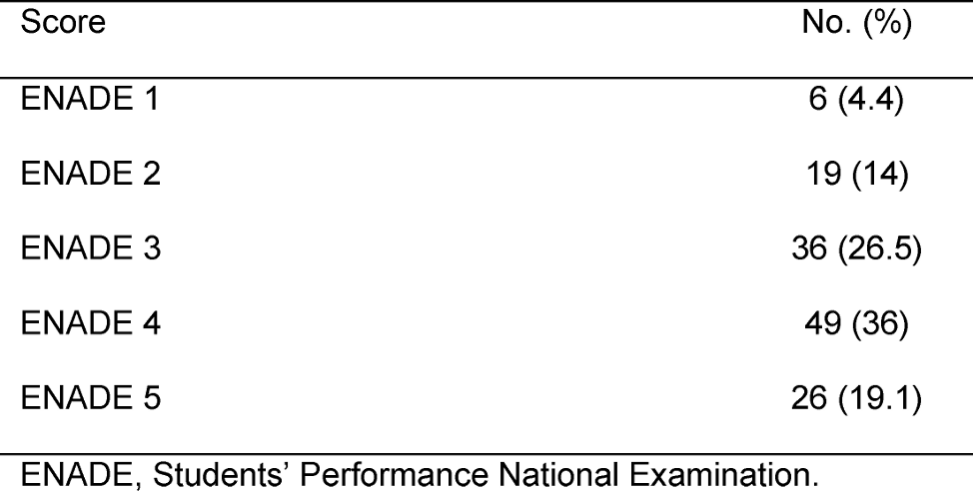
Medical Schools’ Performances According to ENADE 2010.

**Table 2:**
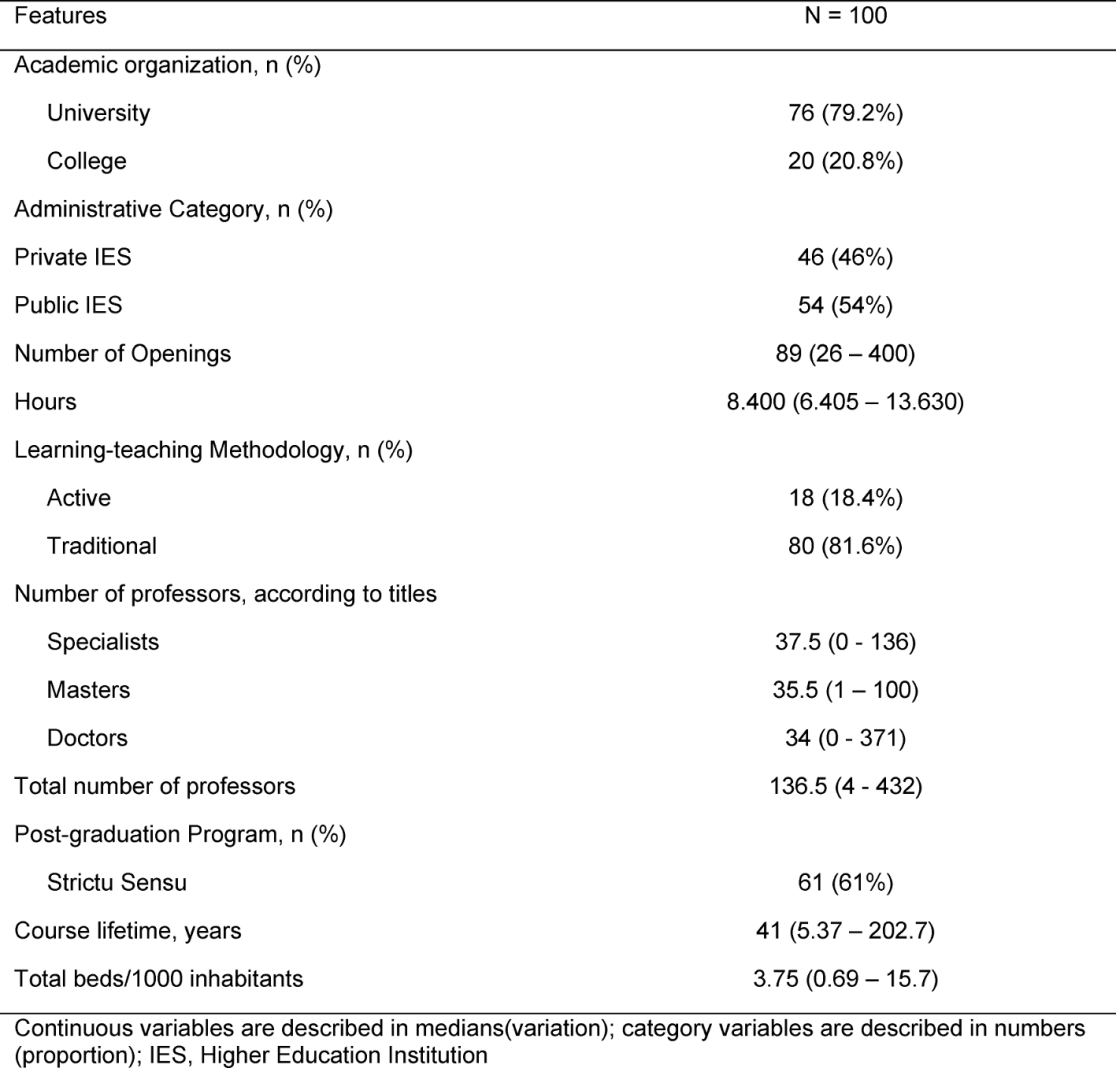
Fundamental Features of the Analyzed Sample.

**Table 3:**
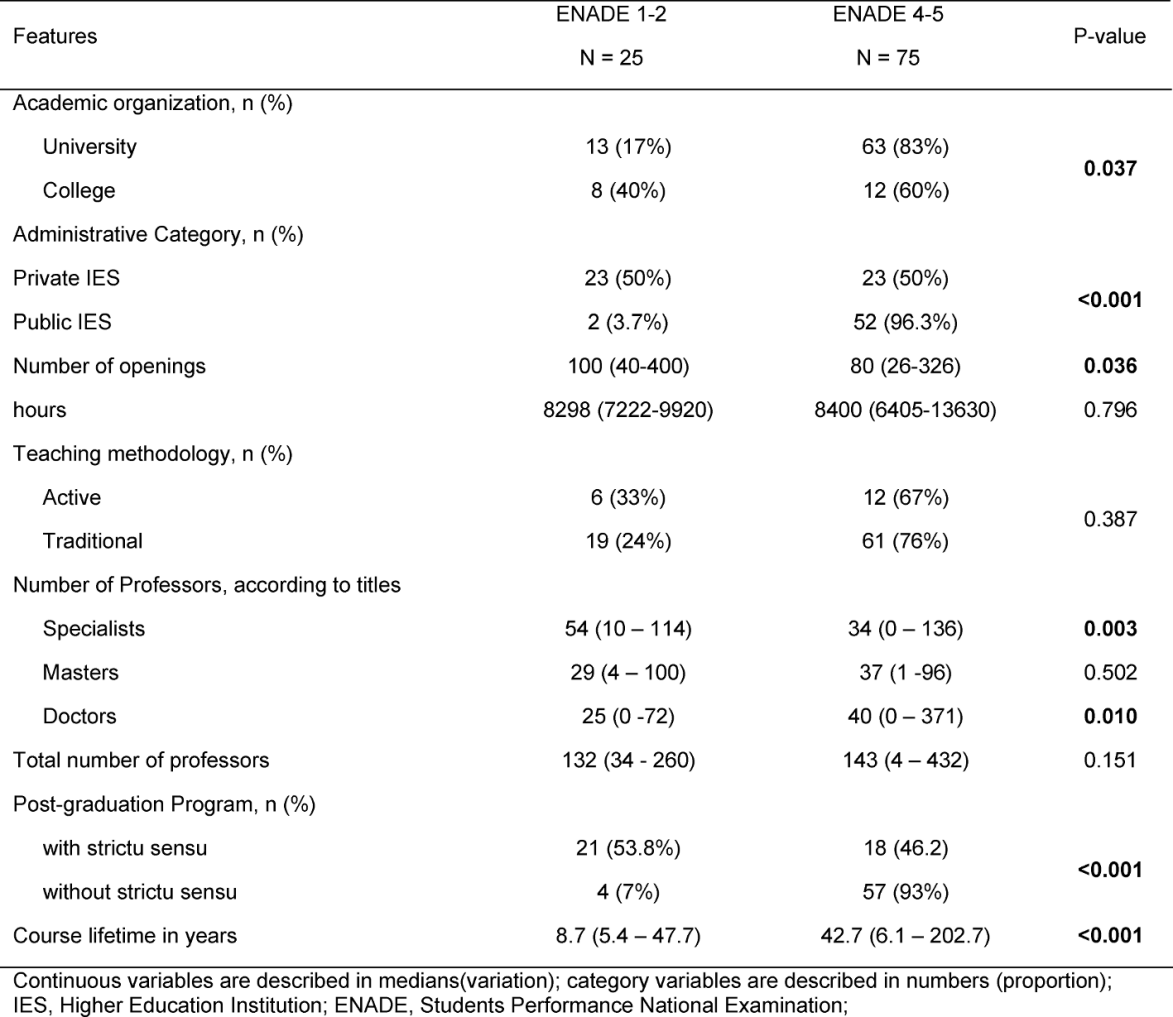
Univariate Comparative Analysis between IES with ENADE 1-2 and ENADE 4-5.

**Table 4:**
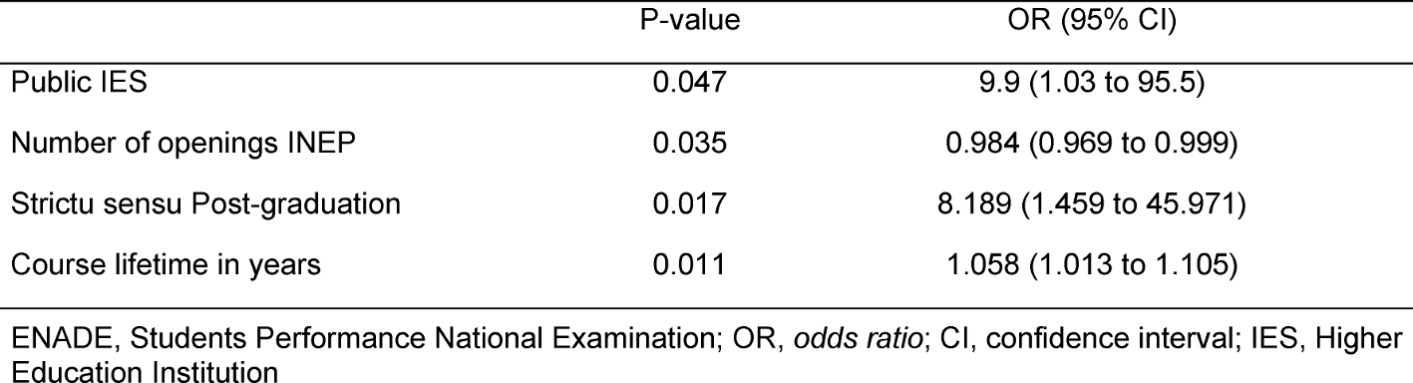
Multivariate Analysis of factors associated to ENADE 4-5 performance.
